# A Thorough Analysis of Changing Antibiotic Resistance in Dental Practice: A Narrative Review

**DOI:** 10.7759/cureus.103939

**Published:** 2026-02-19

**Authors:** Sumeet Agarwal, Laresh Mistry, Ekta Jangid, Shefali Talekar, Saba Kondkari, Rupesh R Raut

**Affiliations:** 1 Prosthodontics, Bharati Vidyapeeth (Deemed to be University) Dental College and Hospital, Navi Mumbai, IND; 2 Pediatric and Preventive Dentistry, Bharati Vidyapeeth (Deemed to be University) Dental College and Hospital, Navi Mumbai, IND; 3 Private Practice, Bharati Vidyapeeth (Deemed to be University) Dental College and Hospital, Navi Mumbai, IND; 4 Conservative Dentistry and Endodontics, Bharati Vidyapeeth (Deemed to be University) Dental College and Hospital, Navi Mumbai, IND; 5 Dentistry, Bharati Vidyapeeth (Deemed to be University) Dental College and Hospital, Navi Mumbai, IND; 6 Prosthodontics and Crown and Bridge, Dr. G.D. Pol Foundation YMT Dental College and Hospital, Navi Mumbai, IND

**Keywords:** antibiotic resistance, antimicrobial stewardship, dental practice, evidence-based prescribing, public health

## Abstract

Antibiotic resistance (ABR) is now a global health issue with major significance in dental practice. While dentistry accounts for only a small percentage of total antibiotic prescriptions, its misuse and overuse are major forces behind the development of resistance. This review extensively summarizes the changing trends in ABR in dental practice, highlighting the contributions of overprescription, self-medication, the absence of standardized guidelines, and educational deficiencies among dentists. The implications of ABR include inferior treatment outcomes and enhanced risks to public health through the dissemination of resistant genes among microbial populations. Possible solutions include antimicrobial stewardship (AMS) programs, new diagnostic technologies, and public and professional education. Special focus is placed on the adoption of evidence-based prescribing, minimization of prophylactic misuse, and utilization of antibiotic alternatives in non-systemic dental infections. Innovations in prescription labeling and awareness campaigns are also proposed as measures to encourage rational antibiotic use.

In the Indian context, this review draws attention to local disparities in antibiotic misuse and urges specific interventions. Finally, preventing ABR in dentistry requires a multifaceted response involving technological innovation, policy change, and educational efforts to maintain the efficacy of antibiotics for generations to come.

## Introduction and background

An antibiotic is described as “a substance produced by microorganisms that can act on other microorganisms (or living cells) by inhibiting their growth or destroying them (antibiotic action)” [1]. The name, which comes from the Greek and means “opposite life,” is actually used to describe medications that may either halt or slow the development of bacteria by blocking a certain metabolic pathway that is crucial to the bacteria. Antibiotic resistance (ABR) has come to be one of the most serious global public health challenges. While dentistry represents a smaller proportion of antibiotic prescriptions than medicine in general, misuse of antibiotics in dental treatment is a key factor in resistance development. In this review, the etiology and implications of ABR in dentistry are discussed, focusing on over-prescription, educational deficits, and the increasing relevance of antibiotic stewardship. Antibiotics are one of the most widely used and abused groups of drugs. In developing nations, antibiotic overuse is common [2]. Greater utilization of antibiotics beyond appropriate indications can be termed over-prescription, inappropriate prescribing, prescribing medication that is not consistent with guidelines, and patients not using the prescribed treatment [3]. Inappropriate consumption of antibiotics may enhance ABR and reduce the effectiveness of antibiotics employed to cure bacterial infections.

A narrative literature review was used for this investigation. To find pertinent papers on ABR in dental practice, an electronic search was conducted utilizing databases such as PubMed, Scopus, Google Scholar, and Web of Science. ABR, antimicrobial resistance (AMR), dental antibiotic prescription, antibiotic abuse in dentistry, and antibiotic stewardship in dentistry were among the keywords used in different combinations. English-language articles that addressed the causes, contributing factors, and consequences of resistance in relation to the prescription of dental antibiotics were included. The inclusion of original research articles, reviews, clinical guidelines, and policy articles was made possible by the lack of strict restrictions on study design. In order to find further pertinent material, the reference lists of the selected papers were also manually examined. The included studies were qualitatively synthesized, with a focus on patient-related factors, over-prescription patterns, dental practitioners' knowledge gaps, and the role of antibiotic stewardship in reducing resistance. Risk of bias assessment and meta-analysis were not carried out because this was a narrative review; instead, the results were interpreted descriptively to provide a thorough picture of the changing problem of ABR in dentistry.

## Review

Drivers of ABR in dental practice

Dentists prescribe antibiotics prophylactically or to treat infections, but research indicates that a large percentage of these prescriptions are not required. Tarun and Sonal (2024) studied the prescribing practices of dental students in India and found that 42% of the prescriptions were not evidence-based. Likewise, Iyer et al. (2025) found that a lack of knowledge regarding mechanisms of resistance leads to rampant overprescription in South Asia [4,5]. Self-medication with antibiotics is also a driver of resistance. Eeman et al. (2024) discovered that patients often use expired antibiotics to self-medicate for dental pain, resulting in inadequate dosages and the development of resistance. This practice is especially alarming in areas where healthcare access is poor [6]. The absence of standardized prescribing procedures is a significant issue that contributes to resistance. Yaqoob et al. (2024) observed that dentists’ use of antibiotics to treat children's dental infections is inconsistent, with the majority using broad-spectrum antibiotics needlessly [7]. Resistance has made certain infections, including periapical abscesses and odontogenic cellulitis, more challenging to treat. A recent scoping review highlights that while prophylactic antibiotics can reduce surgical site infections in selected oral and maxillofacial procedures, their benefits are highly procedure-specific, and prolonged or routine use offers no additional advantage in many cases. These findings reinforce the need for judicious, short-course, evidence-based antibiotic prophylaxis to minimize unnecessary exposure and help combat the growing threat of antibiotic resistance in dental and maxillofacial practice [8]. The collective effect of ABR in dentistry goes beyond the individual patient. Resistance in oral bacteria can act as a reservoir for genes that can spread to other bacterial populations, thereby fueling the public health problem [9].

Antibiotic stewardship in dental practice

The “coherent set of actions which promote the responsible use of antimicrobials” is known as antimicrobial stewardship (AMS) [10]. With several studies already attempting to conduct trials on various strategies to promote appropriate dental antibiotic usage, it is evident that AMS integration into oral health practice is necessary. According to a handbook on AMS, identifying opportunities and obstacles, creating solutions to effect change, and having the ability to monitor results are all essential components of creating a successful AMS protocol [11]. Clindamycin is often prescribed by dentists in the US and Canada, and it is also included as an option for patients who are allergic to penicillins in both nations’ guidelines. Compared to amoxicillin or metronidazole, clindamycin is more strongly associated with *Clostridioides difficile* infection [12]. Delabeling could be one strategy to reduce incorrect medication use and improve patient safety, considering the possibility of referring individuals for penicillin allergy evaluation. Studies of context-dependent factors for each location are essential when setting up AMS in dental practice [13]. Education is also crucial in controlling ABR. Laverty et al. (2024) highlighted the requirement for continuing education courses to enhance prescribing habits among dentists. Such courses have been promising in minimizing overprescription [14]. Evidence from systematic reviews indicates that digital antimicrobial stewardship interventions, particularly clinical decision support systems, are effective in reducing overall antibiotic use and improving prescribing appropriateness in hospital settings. These findings emphasize the growing role of technology-assisted stewardship in optimizing antibiotic utilization and highlight its potential to mitigate inappropriate prescribing practices that contribute to the development and spread of antibiotic resistance [15]. Thabit et al. (2024) promoted a more effective partnership between pharmacists and dentists to oversee and control antibiotic prescriptions. Their research illustrates pharmacists’ roles in detecting inappropriate prescriptions and suggesting alternatives [16].

Innovations to combat ABR

Rapid Diagnostic Tools

One area to watch is the creation of rapid diagnostic tools for detecting bacterial pathogens and resistance profiles. These technologies allow dentists to personalize antibiotic therapy based on patients’ needs, decreasing unnecessary prescriptions [17].

Alternatives to antibiotics: Investigational efforts regarding non-antibiotic treatments, including antimicrobial peptides, are becoming increasingly popular. These agents hold potential for controlling dental infections without contributing to the development of resistance [18]. A large number of dental pain cases are pulp infections, which require operative management by the dentist instead of antibiotics [19]. Clinical situations that demand empiric antibiotic therapy are few and limited to oral infections that present with evidence of systemic dissemination (e.g., fever, lymphadenopathy, and lockjaw). Consistent with this, not all odontogenic infections require antibiotics and should not rely on them as a replacement for removal of the infection’s cause [20]. Antibiotics are unable to reach the pulp and eradicate the germs because the pathophysiology of pulp disease compromises blood flow to the root canals. The only way to eliminate the infection and relieve the symptoms is through endodontic therapy [21]. Nonetheless, antibiotics are still prescribed by dentists worldwide for localized illnesses that do not involve systemic manifestations. Systemically administered antibiotics are not advised, even for peri-implantitis and periodontitis [22].

Penicillin, erythromycin, cefadroxil, metronidazole, tetracyclines, and clindamycin are all effective oral antibiotics for odontogenic infections. The severity of the infection and the most common bacterial species dictate the type of antibiotic, its combination, and dosage [23]. However, the most frequently recommended antibiotics in dentistry are amoxicillin and amoxicillin + clavulanic acid [24]. Standardization in prescription labeling is crucial for patient safety, particularly for oral liquid dosages, where mistakes in dosing can have major repercussions.

To eliminate misunderstanding caused by non-standard volume units like teaspoons or tablespoons, the National Council for Prescription Drug Programs (NCPDP) has released a white paper that offers specific recommendations for improved dosage clarity. The report advocates for the sole use of “mL” as a standard unit [25]. The use of leading zeros for doses below one and the avoidance of trailing zeros following decimal points were also stressed, thereby decreasing tenfold dosing mistakes. In addition, the provision that all oral liquid medications dispensed must be delivered with calibrated dosing devices compatible with the labeled directions is extremely important. These suggestions are particularly pertinent in dental practice, where antibiotics are commonly used in oral liquid form for the pediatric or geriatric patient population. Consistent labeling and patient information can then act as core instruments in decreasing dosing errors and supporting AMS activities in dental practice.

Table 1 shows an overview of the NCPDP’s guidelines for labeling oral liquid medications.

**Table 1:** An overview of the NCPDP's guidelines for labeling oral liquid medications NCPDP, National Council for Prescription Drug Programs

Recommendation	Rationale	Implementation Notes
1. Standard unit is "mL"	Avoids confusion with more than one unit such as tsp, tbsp, cc, etc.	Avoid “cc,” “ml,” or spelled-out forms; use only “mL”
2. Leading zeros for decimals (e.g., 0.5 mL)	Avoids overdose when reading decimals wrongly	Never use trailing zeros (e.g., 2.5 mL, not 2.50 mL)
3. Always discard dosing devices labeled in mL	Aligns label with real dosing tools to avoid measurement mistakes	Show calibrated tools or make patient access available
4. Provide pharmacy staff and patient education	Facilitates consistent and safe dosing instruction use	Utilize brochures, face-to-face counseling, and standard units in communication

Better prescription standards are increasingly identified as being of paramount importance in improving health literacy and medication safety, especially considering the widespread misuse and misunderstanding of medications. In dentistry, where empirical and unmonitored antibiotic prescribing is common practice, standardized labeling and explicit instructions may be instrumental in preventing improper antibiotic use. According to Kennedy and DiParlo (2009), standardized prescription and container label structures - particularly those using FDA-approved indications - can lead to substantial decreases in variability in drug interpretation and enhance patient compliance [26]. ABR is a serious threat not only to overall medical practice but also to the field of dentistry. A review highlights that overprescription, nonadherence to clinical guidelines, and empirical treatment approaches are key drivers of AMR in dental clinics [27]. Dental caries are most often linked with bacterial infections, and knowledge of the antibiotic susceptibility of these bacteria is crucial for successful treatment. A Nepalese study investigated ABR patterns among aerobic bacteria cultured from patients with dental caries.

The most commonly isolated pathogens were *Streptococcus mutans*, *Staphylococcus aureus*, and *Lactobacillus* species - the primary oral infecting pathogens. Routine antimicrobial susceptibility testing in dental practice is required to inform treatment decisions and prevent unnecessary prescriptions [28]. ABR has progressively developed into a public health crisis, fueled by the extensive overuse of antibiotics in human medicine, veterinary medicine, and agriculture. The irrational and unregulated use of antimicrobials, coupled with low levels of infection control practices and inadequate surveillance systems, has resulted in the development of multidrug-resistant organisms that are increasingly challenging to treat.

This escalating resistance crisis threatens to undermine routine medical and dental procedures, as formerly treatable infections become life-threatening. Compounding the issue is the stagnation in the development of new antimicrobial agents, leaving the medical community with limited therapeutic options. To offset this threat, there needs to be a unified global strategy - imposing stricter regulations on antibiotic dispensation, advocating for AMS, increasing monitoring of resistance, and creating public awareness about the judicious use of antibiotics [29].

In most cases, not all tooth extractions require antibiotics. For instance, dentists may mistake surgical trauma symptoms (such as postoperative pain, edema, and trismus) for postsurgical infections. Instead of utilizing antibiotics to treat them, anti-inflammatory and antiseptic therapies should be used [30]. Furthermore, alveolar osteitis, also known as dry socket, is not treated with antibiotics, since it is an extraction-related complication that causes a patch of exposed bone without a blood clot, thereby delaying the healing of the extraction site after exodontia. In addition to painkillers and patient reassurance, treatment options, instead of antibiotics, include saline irrigation or chlorhexidine mouthwash [31].

Antibiotic therapy is not required for most endodontic infections in immunocompetent individuals that do not involve systemic manifestations, such as irreversible pulpitis, pulp necrosis, acute apical periodontitis, chronic apical abscesses, or acute apical abscesses. Root canal treatment, drainage for localized fluctuant edema, and pain medication for symptomatic cases can all be effective forms of treatment. Therefore, systemic antibiotic therapy is usually not necessary [32]. When there is no blood flow to the affected area, as in pulp necrosis, systemic antibiotics are not recommended, since the antibiotic would not be able to efficiently reach the affected area [32]. In addition, the use of antibiotics to control pain after endodontic treatment is not appropriate, since the pain is the result of an inflammatory process, not an infectious process. Pain can be well controlled with opioid analgesics, including hydrocodone or oxycodone, or nonsteroidal anti-inflammatory drugs (NSAIDs), including ibuprofen or diclofenac [33]. Antibiotics are routinely prescribed in dental practice; however, a large share of these treatments is unnecessary, leading to an escalating issue of AMR.

The inappropriate use is most apparent in endodontic and oral surgical cases, in which antibiotics tend to be prescribed “just in case” or because of patient expectations, rather than because of clinical necessity. The review also reported significant variation in prescribing worldwide, with variability too often associated with the absence of current, evidence-based guidelines and limited availability of continuing education. In addition, delayed prescribing techniques - where patients are prescribed antibiotics but told to delay their use - were underused, even though they were effective at lowering consumption. The results highlight the pressing need for enhanced diagnostic procedures, improved clinical guidelines, and focused stewardship interventions appropriate to dental practice.

Education, audit, and feedback processes should improve prudent prescribing practices and reduce dentistry's contribution to the problem of ABR [34]. Dental radiography, the insertion of removable appliances, bleeding from damage to the lips or oral mucosa, anesthetic injections via noninfected tissue, the loss of primary teeth, and other procedures that do not involve the gingival tissue or periapical region do not require antibiotic prophylaxis for infective endocarditis (IE) in high-risk patients [35]. Preventive medications should not be administered to all individuals with prosthetic joints in order to avoid infections. Only in specific clinical settings, such as when treating a patient with a complicated history of prosthesis-related issues, should prophylaxis be considered when the patient’s invasive dental procedure poses a significant medical risk. After consultation with the patient’s orthopedic physician, prophylaxis is advised when risk factors are present [36].

Regional Focus: India’s ABR

Due to its large population and unequal access to healthcare, India faces unique difficulties related to ABR. This imbalance underscores the importance of targeted interventions [37]. State-level research is required to determine the characteristics that affect inappropriate use at the provider, patient, and health system levels, given the significant differences observed across states. Best practice examples may include states where the appropriate use of antibiotics has increased over time [38].

Frequent antibiotic use: immune dysregulation and systemic consequences

The human immune system relies on a delicate balance between microbial exposure, tolerance, and regulation. Frequent or inappropriate antibiotic use disrupts this equilibrium, leading to immune sluggishness - a state characterized by diminished responsiveness and regulatory imbalance [39]. Antibiotics indiscriminately eliminate both pathogenic and beneficial microbiota, particularly in the gut, which plays a central role in immune education and homeostasis. Loss of microbial diversity impairs the maturation of T regulatory cells, reduces mucosal immunity, and alters cytokine profiles, ultimately leading to immune hyporesponsiveness and heightened susceptibility to infections, allergies, and autoimmune disorders [40].

Beyond the immune system, dysbiosis caused by antibiotics impacts metabolic, neurological, and endocrine functions. Microbial metabolites, such as short-chain fatty acids (SCFAs), are crucial for gut-brain communication, glucose homeostasis, and epigenetic regulation. Their depletion can contribute to conditions such as metabolic syndrome, mood disorders, and even neurodegeneration [41]. At the cellular level, repeated antibiotic exposure induces mitochondrial stress, oxidative damage, and impaired signaling pathways. Some of these changes - particularly in immune and epithelial cells - may be irreversible due to epigenetic modifications and stem cell exhaustion [42]. Therefore, frequent antibiotic use not only diminishes acute immune function but also sets in motion systemic dysfunctions that may persist or worsen over time. Prudent, evidence-based antibiotic prescribing is essential to preserve both immediate and long-term immunological and systemic health.

Preventing antibiotic use in children: a public health imperative

The unnecessary use of antibiotics in children is a pressing global health concern that accelerates the development of AMR. Pediatric populations are frequently prescribed antibiotics for viral infections such as the common cold, otitis media, and bronchitis - conditions in which antibiotics are ineffective [43]. Preventing such misuse demands a multifaceted approach rooted in education, surveillance, and stewardship.

First, parental and provider education is crucial. Many caregivers expect antibiotics during pediatric visits, which contributes to inappropriate prescribing [44]. Clear communication about the viral nature of most childhood infections and the risks of AMR can reduce pressure on clinicians to prescribe unnecessarily. Second, robust AMS programs tailored for outpatient pediatric care have shown significant success. These programs provide clinicians with evidence-based guidelines and decision-support tools, reducing inappropriate prescriptions by up to 36% [45]. Third, enhancing vaccination coverage indirectly reduces antibiotic use. Vaccines such as the pneumococcal conjugate vaccine decrease the incidence of bacterial infections, thereby reducing the need for antibiotics [46]. Finally, policy-level interventions - including stricter prescription regulations and public awareness campaigns - can further curb misuse.

To protect future generations, antibiotic prescribing in children must be restricted to evidence-based, clinically justified scenarios. Without urgent, coordinated action, the post-antibiotic era threatens to become a reality.

## Conclusions

A diversified strategy is necessary to counteract the growing threat of ABR in dentistry. It is critical to strengthen antibiotic stewardship initiatives, particularly by expanding their outreach to marginalized and rural regions. At the same time, the accuracy of antibiotic prescriptions can be greatly improved by incorporating rapid diagnostic technology into standard dental procedures. To guarantee consistency in practice, legislative changes that establish and implement uniform principles nationwide are equally crucial. Public education initiatives should also be given top priority in order to increase awareness of the risks associated with self-medication and the excessive use of antibiotics. Modern dental treatment is seriously threatened by ABR; however, the dental profession can play a critical role in reducing this developing issue by implementing a multidisciplinary strategy that includes education, technology, and policy reform.
